# Combination of error-prone PCR (epPCR) and Circular Polymerase Extension Cloning (CPEC) for improving the coverage of random mutagenesis libraries

**DOI:** 10.1038/s41598-024-66584-y

**Published:** 2024-07-10

**Authors:** Natalia Ossa-Hernández, Luis Fernando Marins, Daniela Volcan Almeida

**Affiliations:** 1 Departamento de Biología, Universidad del Valle (UV), Cali, Colombia; 2 Laboratório de Biologia Molecular, Instituto de Ciências Biológicas, Universidade Federal do Rio Grande (FURG), Rio Grande, Brazil; 3 Instituto de Biologia, Universidade Federal de Pelotas - UFPEL, Campus Universitário Capão do Leão s/n, Pelotas, RS 96160-000 Brazil

**Keywords:** DNA recombination, Gene amplification, Molecular cloning, Random mutations, Variant genes, Expression systems, Synthetic biology

## Abstract

Random mutagenesis, such as error-prone PCR (epPCR), is a technique capable of generating a wide variety of a single gene. However, epPCR can produce a large number of mutated gene variants, posing a challenge in ligating these mutated PCR products into plasmid vectors. Typically, the primers for mutagenic PCRs incorporate artificial restriction enzyme sites compatible with chosen plasmids. Products are cleaved and ligated to linearized plasmids, then recircularized by DNA ligase. However, this cut-and-paste method known as ligation-dependent process cloning (LDCP), has limited efficiency, as the loss of potential mutants is inevitable leading to a significant reduction in the library’s breadth. An alternative to LDCP is the circular polymerase extension cloning (CPEC) method. This technique involves a reaction where a high-fidelity DNA polymerase extends the overlapping regions between the insert and vector, forming a circular molecule. In this study, our objective was to compare the traditional cut-and-paste enzymatic method with CPEC in producing a variant library from the gene encoding the red fluorescent protein (DsRed2) obtained by epPCR. Our findings suggest that CPEC can accelerate the cloning process in gene library generation, enabling the acquisition of a greater number of gene variants compared to methods reliant on restriction enzymes.

## Introduction

Proteins are biomolecules with significant potential for applications in research, the food and pharmaceutical industries^1^, and disease treatment^2^. Their amino acid sequences are determined by the information encoded in the genes. These sequences evolve due to spontaneous mutations, which are naturally selected by the environment or other factors. Numerous technologies have been developed to study protein evolution in the laboratory. These aim to accelerate mutational rates, generate a vast array of variants, and facilitate the selection of proteins with desired characteristics. Different alterations to the polymerase chain reaction (PCR) method have made both site-specific and random mutagenesis more accessible. This has improved enzyme stability across broader pH and temperature ranges and increased tolerance to various organic solvents. There are many specific methods for protein engineering, which can be broadly categorized into two main groups: those based on the rational design of protein modifications and combinatorial methods that introduce changes randomly (For review see^3^).

Random mutagenesis, for example, is a technology capable of generating high diversity from a single gene that involves the creation of libraries consisting of large numbers of genetic variants, where each member can be isolated and individually assessed concerning the genotype–phenotype relationship^4^. Genetic variants can be obtained from methods such as error-prone PCR (epPCR). This last methodology, developed by Leung et al.^5^, uses a low-fidelity DNA polymerase that, under certain conditions, can introduce random mutations during PCR amplification of a target gene. The products of these mutagenic PCRs must then be linked to plasmid vectors and used to produce gene libraries. Although epPCR can generate an extremely large number of mutated gene, there is a limitation in ligating mutated PCR products into the plasmid vectors used to generate the libraries. The practical utility of any cloning method is based on its reliability, cost, or efficiency under optimal conditions. Methods that are easier to monitor and optimize are the most reliable^6^. The earliest cloning method used was Ligation-Dependent Cloning Process (LDCP) This method is used for cloning mutant fragments obtained by epPCR, for this the primers used are designed to include artificially specific restriction enzymes recognition sites that are compatible with the restriction sites present in the plasmid used. Thus, the PCR products must be cut with the chosen enzymes and then ligated to the plasmid previously linearized with the same enzymes. The cohesive termini will allow the recircularization of the vector by the action of a DNA ligase. This cut-and-paste process has limited efficacy and causes a significant reduction in the scope of the library since the loss of potential mutants is unavoidable.

Interesting alternatives to the LDCP to clone PCR products includes methodologies free of restriction enzymes and ligases^7–9^, and the the Circular Polymerase Extension Cloning (CPEC) method developed by Quan and Tian^10^. The last one consists of a reaction where a high-fidelity DNA polymerase extends the overlapping regions between the insert and vector to form a circular molecule. CPEC offers significant advantages in terms of simplicity, efficiency, and economy. It has even been suggested to produce gene libraries^11^. Compared to techniques reliant on restriction enzymes, the use of CPEC in creating gene libraries may accelerate the cloning process and produce a higher number of gene variations. In this case, our goal was to evaluate the effectiveness of CPEC vs the conventional cut-and-paste enzymatic technique for generating a variant library from the epPCR-obtained gene encoding the red fluorescent protein (DsRed2).

## Material and methods

### Step 1—Obtaining the mutant insert by error-prone PCR and the control insert

**Figure 1:**
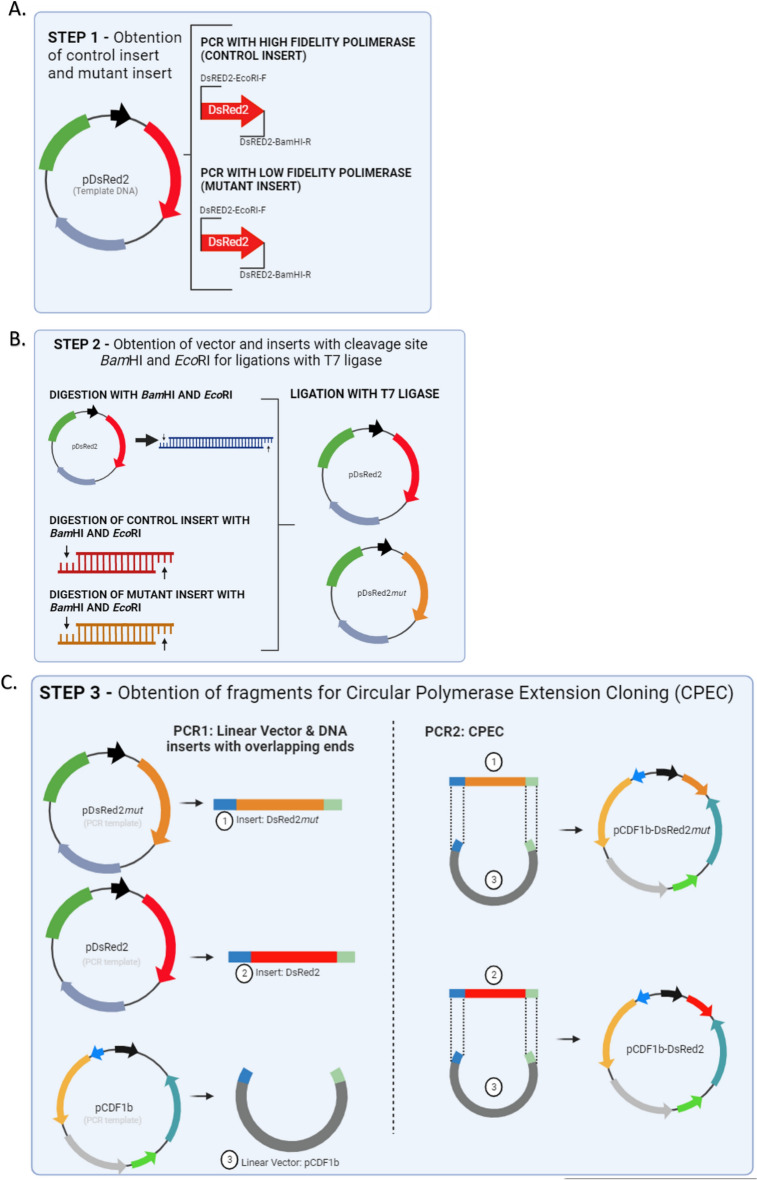
Graphic representation of the main methodological steps for comparing the Ligation-Dependent Process Cloning method (LDPC) and Circular Polymerase Extension Cloning (CPEC). (**A**) Step 1 – Obtaining the Mutant Insert by Error-Prone PCR and the Control Insert. The DsRed2 gene was isolated from the plasmid pDsRed2 through error-prone PCR using specific primers and conditions, resulting in the mutant insert. The control insert was also isolated from the plasmid pDsRed2, but a high-fidelity polymerase was used. (**B**) Step 2—Ligation-dependent process cloning. A vector was prepared by cleaving the pDsRed2 plasmid with BamHI-HF and EcoRI-HF enzymes, followed by digestion of all fragments (control insert and mutant insert from Step 1) using the same restriction enzymes, and ligation reactions were performed using T7 ligase. (**C**) Step 3—Circular Polymerase Extension Cloning – CPEC. The mutant insert, along with the control, was amplified via PCR, quantified, and cloned into the pCDF1b (GenBank Accession Number OR900361.1) expression vector using CPEC with overlapping primers.

The DsRed2 gene was isolated using the plasmid pDsRed2 (Clontech, Cat. No. 632404, UniProt Q9U6Y8) as a template (Fig. 1A). Error-prone PCR of the DsRed2 gene was performed using the GeneMorph® II Random Mutagenesis kit, following the manufacturer’s protocol The primers DsRed2-EcoRI-F and DsRed2-BamHI-R (Table 1) were used and the PCR conditions included one cycle at 94 °C for 2 min, followed by 30 cycles at 94 °C for 15 s, 68 °C for 30 s, and 72 °C for 60 s, with a final elongation step at 72 °C for 5 min. The products of error-prone PCR are referred to as the mutant insert in this text. The DsRed2 gene without mutations (Fig. 1A) was amplified using the same primers and high-fidelity polymerase (TAKARA LA Taq DNA, Clontech Cat. No. RR002A) as a control for the procedure (referred to as the control insert). The PCR conditions for this were 94 °C for 2 min, followed by 30 cycles of 94 °C for 15 s, 60 °C for 30 s, 72 °C for 2 min, and a final cycle at 72 °C for 5 min. After PCR, the amplicons were verified on 1% agarose gel electrophoresis and purified using the Illustra GFX® PCR DNA and Gel Band Purification Kit (GE Healthcare).

**Table 1:** The specific primers used to amplify the genes and vectors.

Primer	Sequence 5′- 3′
DsRed2-EcoRI-F	CGGCCGCGACTCTAGAATTCCAAC
DsRed2-BamHI-R	ACCGGTACCCGGGGATCCTCTA
Mut/DsRed2-F	ATGGCCTCCTCCGAGAACGTCATCA
Mut/DsRed2-R	CAGGAACAGGTGGTGGCGGCCCTCG
PCDF-F*	GCCGCCACCACCTGTTCCTGCTCGAGTCTGGTAAAGAAAC
PCDF-R*	ACGTTCTCGGAGGAGGCCATGTCGACCTGCAGGCGCGCCG
DsRed2-Seq- F	GCTATGACCATGATTACGCCAAGC
DsRed2-Seq-R	GGGCCCGTACGGCCGACTA
PCDFBGL-Seq -F	CTGACGCGTTACCGGAAGCAGTGTGACCGTGT
PCDFBGL-Seq -R	CACGAATTCTCGACGCTCTCCCTTATGCGACT

The highlighted sequences indicate cleavage sites or oligonucleotides with overlapping sequences (*).

### Step 2—Ligation-dependent process cloning

Initially, the pDsRed2 plasmid was cleaved (Fig. 1B) using the enzymes BamHI-HF (New England Biolabs, Cat. No. R3136) and EcoRI-HF (New England Biolabs, Cat. No. R3101) to get the vector. Afterwards, BamHI-HF and EcoRI-HF restriction enzymes were used to digest all fragments (control insert and mutant insert – Step 1). This digestion took place over an incubation time of 2 h at a temperature of 37 °C. The enzymes were inactivated for 20 min at 65 °C. Digested fragments were quantified on the Qubit fluorimeter (Life Technologies, Brazil) using the Quant-iT dsDNA BR Assay kit (Invitrogen, Brazil). A 1:1 ratio was used for the ligation reactions. The vector (pDsRed2) was at a concentration of 81.7 ng/µL and the inserts were at a concentration of 84.1 ng/µL. The ligation using the T7 ligase (New Englands, Biolabs Cat. No M0318) was carried out according to the manufacturer’s protocol and was conducted in triplicate.

#### Bacterial transformation for T7 ligation products

A total of the 1 µL of product from each ligation was transformed into 40 µL of electrocompetent *Escherichia coli* TOP 10 bacteria (0.2 cm cuvette, 2.5 kV/cm, 25 µF, 200 Ω, 1 pulse) using the Gene Pulser Xcell™ electroporation system (BioRad). The cells were grown in 480 µL of SOC medium (2% tryptone, 0.5% yeast extract, 0.05% NaCl, 2.5 mM KCl, 20 mM glucose) for 1:30 h at 37 °C with constant shaking at 243 g in a Stuart Shaking incubator SI500 orbital shaker (Stuart, Brazil). After incubation, the inoculants were seeded in plates containing Luria Bertani (LB) agar medium and antibiotic spectinomycin (100 µg/mL) and incubated for 16 h at 37 °C. The bacteria transformed with the product of each ligation were screened for strong fluorescence using the Safe Imager™ 2.0 Blue Light Transilluminator (Invitrogen) with excitation at 470 nm. The plates obtained were photographed and the total number of colonies on each plate was determined. The plates for the controls and mutants were quantified using microscopy and counted with ImageJ software.

### Step 3—Circular polymerase extension cloning—CPEC

We utilized the construct that was obtained and chosen from Step 2 (pDsRed*mut*) as a template for the construct that included the mutant insert. PCR reaction was performed using the primers Mut/Dsred2-F and Mut/Dsred2-R (Table 1) and the TAKARA *LA taq* high fidelity DNA polymerase (5U/µL *TAKARA LA Taq,* 10X LA PCR buffer II (Mg^2+^ free, 25 mM MgCl_2_, 0.25 mM dNTP). The PCR conditions were one cycle of 94 °C for 2 min (initial denaturation) followed by 30 cycles of 94 °C for 15 s, 66 °C for 30 s, and 68 °C for 3 min, and a final elongation of 72 °C for 10 min. After PCR, the fragments (hereafter mutant) were quantified using the *Quant-iT dsDNA HS Assay kit* (Invitrogen, Brasil). The same procedure was done in the DsRed2 gene as a control. The mutant gene and the control were cloned into the pCDF1b expression vector (Novagen, Cat. No. 71330-3) (Fig. 1C). The ligation of fragments (DsRed2 and DsRed *mut*) with vector (pCDF1b) was done via CPEC with the primers PCDF-F and PCDF-R (Table 1). These oligonucleotides have an overlapping sequence (bases under-arrayed in the sequence) with the product mutant for CPEC to occur.

The PCR for CPEC was carried out using the TAKARA LA *Taq enzyme* (Clontech Cat. No. RR002A), following the conditions: 94 °C/2 min, 30 cycles of 94 °C/15 s, 63 °C/30 s, 68 °C/4 min and 1 final cycle 72 °C/5 min. The template DNA for the CPEC reaction was the double-stranded fragments of the mutant and the vector pCDF1b was added in a 1:1 ratio. In the first PCR cycle, the fragments are denatured. In the following cycles, the single strands are ringed in the sequence in which they overlap, and it is from this overlap that the fragments extend to form the double strand of the circular plasmid pCDF1b-DsRed2*mut* and pCDF1b-DsRed2, respectively. The fragments were analyzed using 1% agarose gel electrophoresis.

#### Bacterial transformation for CPEC products

The expression vectors produced (pCDF1b-Mutant and pCDF1b-DsRed2) were transformed into electrocompetent *Escherichia coli* BL21-DE3 by electroporation (0.2 cm cuvette, 2.5 kV/cm, 25 μF, 200 Ω, 1 pulse) using the Gene Pulser Xcell™ electroporation system (BioRad). The transformed bacteria were seeded in plates containing Luria Bertani (LB) agar medium and antibiotic spectinomycin (100 μg/mL) and incubated for 16 h at 37 °C. After transformation, bacterial colonies were inoculated into liquid Luria Bertani (LB) medium, using antibiotic spectinomycin (100 μg/mL) as a selective agent, incubated for 16 h at 37 °C with constant shaking at 243 g in a Stuart Shaking incubator SI500 (Stuart, Brazil). Subsequently, the plasmids were purified using the Ilustra™- Plasmid Prep Mini Spin Kit (GE Healthcare). The plates obtained were photographed and the total number of colonies on each plate was determined. The plates for the controls and mutants were quantified using microscopy and counted with ImageJ software.

The selected bacterial colonies were inoculated into liquid Luria Bertani (LB) medium, using the antibiotic spectinomycin (100 μg/mL) as a selective agent, incubated for 16 h at 37 °C with constant shaking at 243 g in a Stuart Shaking incubator SI500 orbital shaker (Stuart, Brazil). Subsequently, the plasmids with mutant and control inserts were purified using the Ilustra™- Plasmid Prep Mini Spin Kit (GE Healthcare). After purification, the plasmids were sequenced using the oligonucleotides PCDFBGL-Seq-F and PCDFBGL-Seq-R (Table 1) to confirm binding using the CPEC and LDCP methodologies.

### Statistical analysis

One-way ANOVA was used for the statistical analysis of the data, with a significance threshold of *p* < 0.05. To make sure the test assumptions were met, tests for homogeneity of variances and residuals’ normality were performed before to the ANOVA. Specifically, Levene’s test was used to assess the homogeneity of variances, and the Shapiro–Wilk test was employed to evaluate the normality of residuals.

## Results and discussion

**Figure 2:**
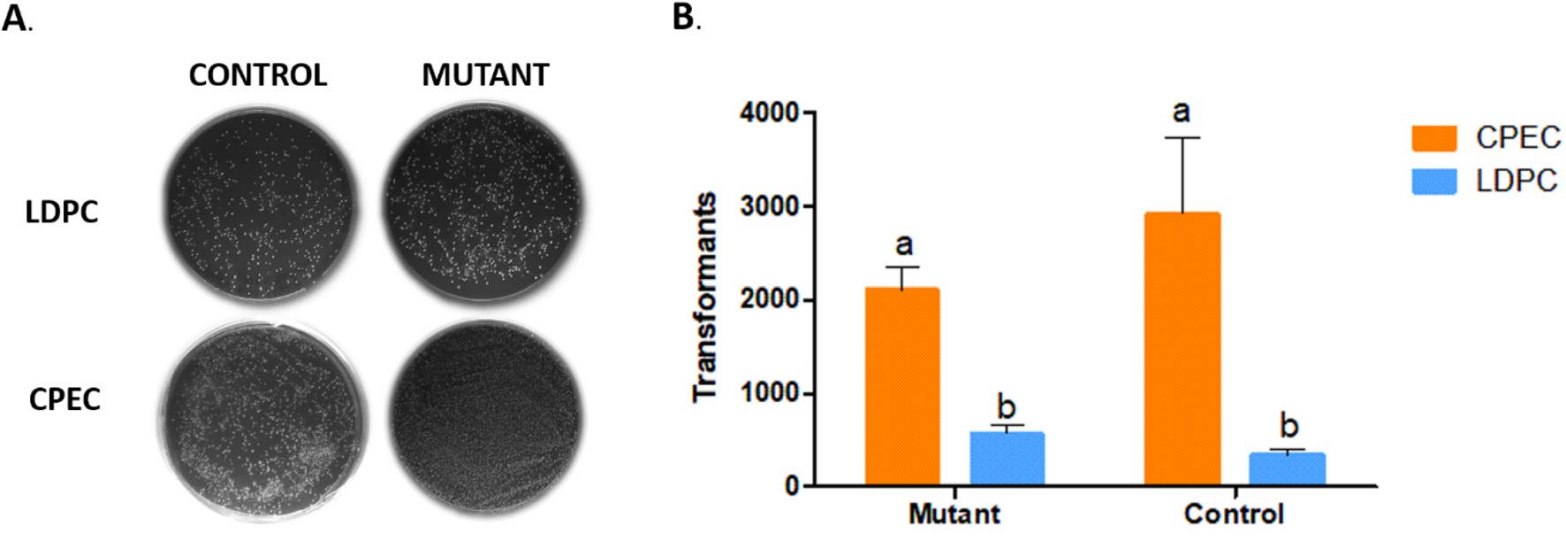
Comparison of colony numbers in Ligation Methods. (**A**) Photos of plates containing the colonies transformed with the vectors pCDF1b-Mutant (Mutant) and pCDF1b-DsRed2 (Control) using the Ligation-Dependent Process Cloning method (LDPC) and Circular Polymerase Extension Cloning (CPEC). (**B**) The average number of colonies obtained by comparing the ligation of the insert (mutant in orange bars and control in blue bars) with the vector (pCDF1b) using the LDPC and CPEC methods. The letters “a” and “b” denote statistically significant differences (*p* < 0.005) in all cases, n = 3.

In order to enhance the efficiency of mutant gene library production, we compared two techniques for linking randomly mutated fragments (generated through epPCR) to the cloning vector. Initially, we employed the gene encoding the red fluorescent protein as a template for random mutagenesis through epPCR. Subsequently, the products epPCR-generated were linked to the cloning vector using two distinct methodologies: the cut-and-paste method, also known as ligation-dependent process cloning (LDCP), and the circular polymerase extension cloning (CPEC) method^10,12^. Both methodologies demonstrated efficiency, with colonies observed on all plates (Fig. 2).

The CPEC-based ligation (Fig. 2B) yielded a higher number of colonies (averaging 2106 colonies for mutant fragments and 2916 for control fragments) compared to the LDCP method (averaging 560 colonies for mutant fragments and 330 for control fragments). These results showed that ligation by CPEC was significantly more efficient than LDCP (*p* < 0.05).

LDCP is a cloning technique that involves linking DNA fragments to the cloning vector through ligation-dependent processes. One advantage of LDCP is its relative simplicity, as it does not require many intricate steps or complex reagents. However, traditional restriction- and ligation-based cloning methods are often associated with low efficiency and time consumption. These methods can be complicated by factors such as incomplete restriction digestion, the effects of DNA methylation, and, at times, limited availability of suitable restriction endonuclease recognition sites. The traditional digestion-ligation method may work better in some circumstances. For instance, when a fragment needs to be released from a vector using restriction digestion and then subcloned into a different vector. But it’s important to remember that, in theory, DNA fragments can also be cloned using a variety of PCR-dependent methods, such as TA cloning^13,14^, Gateway cloning^15^, Gibson assembly cloning^16^, and CPEC^10,11^.

We choose CPEC because it is directional, positional, sequence-independent, and requires only two steps and one enzyme. To do this, we linearize the vector using PCR and create an insert that is complementary to the vector by adding 20 bp (Fig. 1C). In this method, both the linear vector and the insert possess overlapping regions at their respective ends. Following denaturation and annealing, these complementary segments come together to form a hybrid molecule, and the extension occurs as they act as templates for each other, resulting in the formation of a full circular structure with two nicks. This circular construct can then be transformed directly into bacteria without the need for additional purification steps, simplifying the process significantly^10,13^. It is important to note that the observed efficiency of transformation in electroporation in the two methods compared (LDCP and CPEC) may be influenced by the salt concentration in the buffers used^17,18^. Future comparative studies utilizing heat shock transformation, which is less sensitive to ionic strength, could help further validate the observed differences between LDCP and CPEC.

In our study, CPEC not only confirmed the simplicity of the method but also demonstrated its efficiency, thereby enhancing the acquisition of gene libraries. The more clones we obtain, the higher the chance of finding the one with the desired phenotypic trait. The search for mutant genes with new phenotypic characteristics is an approach frequently employed in functional genetics and genetic engineering studies to understand how genes influence organism phenotypes and to develop desired traits in target organisms. The choice between CPEC and LDCP depends on the specific needs of the project and the convenience of the technique in each context. Nonetheless, our preference for CPEC is based on its simplicity, efficiency, and suitability for our research objectives. The field of molecular biology continues to evolve, offering a range of cloning techniques, and researchers should always consider the most appropriate method for their specific applications.

## Conclusion

The binding of variant fragments of the DsRed2 gene using the CPEC methodology is more efficient when employed in the generation of gene libraries mutated by epPCR. The enhanced efficiency of binding variant genes into an expression vector results in a greater number of genes with the potential to exhibit novel phenotypes.

## Data Availability

The data and materials used in this study are available upon request from the corresponding author, D.V.A. The sequence data used in this study are available at https://www.ncbi.nlm.nih.gov for pCDF1b (Accession Number OR900361.1) and at https://www.uniprot.org for the plasmid pDsRed2 (Accession Number Q9U6Y8).
